# Functional, Nutraceutical, and Pharmacological Properties of Black Seed

**DOI:** 10.1002/fsn3.70725

**Published:** 2025-08-19

**Authors:** Muhammad Tayyab Arshad, Sammra Maqsood, Ali Ikram, Muhammed Adem Abdullahi

**Affiliations:** ^1^ Functional Food and Nutrition Program, Faculty of Agro‐Industry Prince of Songkla University Songkhla Thailand; ^2^ National Institute of Food Science and Technology, University of Agriculture Faisalabad Faisalabad Pakistan; ^3^ University Institute of Food Science and Technology, the University of Lahore Lahore Pakistan; ^4^ Department of Food Science and Postharvest Technology Jimma University College of Agriculture and Veterinary Medicine, Jimma University Jimma Ethiopia

**Keywords:** antioxidant, antiviral, food applications, toxicity

## Abstract

Since ancient times, black seed, or *Nigella sativa*, has gained popularity in modern industry and health due to its numerous nutritional and therapeutic applications. Detailed coverage of the botanical description, taxonomy, and phytochemical composition can be seen in this review about the most crucial bioactive compounds, such as thymoquinone, alkaloids, saponins, and fixed oils. This therapeutic plant has undergone numerous studies about its solicitations in treating cancer, its protective effects on the heart, and its antiviral, antibacterial, anti‐inflammatory, antioxidant, and antidiabetic properties. Side by side with its function in conventional medical practices of Ayurveda and Unani, its amalgamation is also specified in the review. This also takes into account the health benefits of black seed, its metabolism, macro‐ and micronutrient composition, and other nutritional considerations. The usages discover the product development potentials within the pharmaceutical and food industries, such as functional foods, health supplements, and cosmetics. Customer attention in natural products has made novel prospects, but side by side with them, there are different concerns about stability, bioavailability, and regulatory problems. To augment the medicinal and economic value of *N. sativa* , there is potential for its genetic development, for more progressive cultivation methods, and for biotechnological policies. Thus, the incorporation of *N. sativa* into current healthcare and industry comprises creative, multidisciplinary investigation.

## Graphical abstract

This review delves into the botanical description, taxonomy, and phytochemical composition of *N. sativa*, specifically focusing on its most significant bioactive compounds, including thymoquinone, alkaloids, saponins, and fixed oils. We explore its extensive medicinal properties, such as anticancer, cardioprotective, antiviral, antibacterial, anti‐inflammatory, antioxidant, and anti‐diabetic effects, while highlighting its integration into traditional medicine systems like Ayurveda and Unani. Furthermore, we analyze its macro‐ and micronutrient composition, metabolism, and health benefits, emphasizing its potential to revolutionize the food and pharmaceutical industries through functional foods, health supplements, and cosmetics.

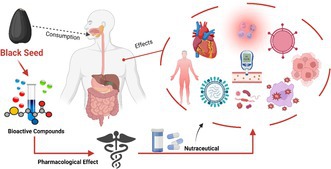

## 1 Introduction

Black seed, or *Nigella sativa* , is well recognized for centuries because of its therapeutic and nutritive prospective. The small flowering plant is native to Africa, the Middle East, and South Asia. Medical, culinary, and even religious solicitations have all advanced significantly from it over the years. Black seed has also been named “the seed of blessing” in Islam, according to Dajani et al. (2018). The herb's history of medicinal usage is demonstrated by indication from the excavation of the herb's seeds from the tomb of Egyptian pharaoh Tutankhamun (Dabeer et al. 2022). *N. sativa* is historically pertinent because of its extensive solicitation in Greco‐Arabic, Unani, and Ayurvedic traditional medicine (Hussain and Hussain 2016). According to Ahmad et al. (2021), the plant has been described by historians like Hippocrates and Avicenna to be of nutritional and medicinal importance. The plant owns various therapeutic benefits, such as digestive and respiratory health. Contemporary scientific investigation has authenticated these assertions by determining pharmacologically active composites in black seed, e.g., thymoquinone, alkaloids, and nigellone (Tavakkoli, Ahmadi, et al. 2017; Tavakkoli, Mahdian, et al. 2017).

Prophet Muhammad purportedly employed black seed as a medication to heal “each disease excluding death” (Hussain and Hussain 2016; Ahmad et al. 2022), and spiritual scriptures frequently reference this fact. An essential characteristic of its history and philosophy is being humiliated by the fact that it is a prophetic practice and Islamic medicine. Expending cures from ancient times also assists to validate the general acceptance and admiration rendered to that tradition as a natural treatment (Dabeer et al. 2022). Foodstuffs and nutrition supplements, and also nutraceutical foodstuffs, typically comprise *N. sativa* due to the long ethnopharmacological practice of the species (Hannan et al. 2021).

Seeds from the *N. sativa* comprise a diversity of bioactive elements, comprising proteins, carbohydrates, fiber, and volatile oils, as well as fixed oils, among others. Most of the seed, roughly 40% of its mass, is the secure oil constituent. This segment is rich in linoleic acid, oleic acid, and polyunsaturated fatty acids, which are all obligatory for the nutraceutical and therapeutic properties of the seed (Mazaheri et al. 2019). Thymoquinone, also found in the seed, is an antioxidant as well as an effective anti‐inflammatory that has now emerged as a prospective candidate in the fight against oxidative stress as well as against long‐lasting diseases (Gholamnezhad et al. 2016). Tavakkoli, Ahmadi, et al. (2017), Tavakkoli, Mahdian, et al. (2017) declare that thymoquinone, a compound found in black seed, has a lengthy array of pharmacological properties, such as antidiabetic, anticancer, and antibacterial activities. Botnick et al. (2012) showed that thymoquinone and supplementary secondary metabolites like alkaloids, saponins, and flavonoids are the reasons for the seed's health benefits. Due to these constituents, black seed can cure numerous diseases, ranging from cardiovascular diseases to neurological conditions (Shabana et al. 2013). The preclinical and clinical applications of *N. sativa* have been the emphasis of a growing body of research over the past few decades. An exciting avenue of research would be the possible application of its supplement in the management of metabolic disorders, infectious illnesses, and chronic inflammatory diseases (Wahab and Alsayari 2023). Astonishingly, though, research into its antimicrobial and antiviral activities in relation to upcoming viral disease pandemics has yielded promising results (Basurra et al. 2021).

*Nigella sativa* has nutritious as well as medicinal values, while the oil and seeds of this plant improve the nutrition and taste of food internationally. Functional foodstuffs, nutraceutical capsules, and fortified oils are particular examples of several products made from black seeds that have newly come into existence due to progress in food technology (Rahim et al. 2022). These developments validate the adaptability of *N. sativa* as a substitute medicine and food additive (Bashir et al. 2021). The capability of *N. sativa* to combine traditional information with modern scientific investigation is the source of its continuing relevance. Its numerous applications as a flavoring, medicine, and nutritional supplement are a pointer of its cultural, historical, and pharmacological importance (Srinivasan 2018). By uniting traditional knowledge with scientific information, scientists have found novel medicinal applications, comprising dietary supplements and medicine preparations (Usmani and Almoselhy 2023). Further investigation is desirable to exploit the usage of *N. sativa* in clinical situations, even though promising potential exists. To exploit its medicinal potential, glitches such as carrying out large‐scale medical trials, isolating bioactive compounds, and establishing dosages need to be addressed (Abbas et al. 2024).

However, it remains a valuable drug in both traditional and complementary medication based on its broad pharmacological and safety contours (Mashayekhi‐Sardoo et al. 2020). It's an outlandish mix that may have wonderful medicinal properties, top‐shelf nutritional properties, and historical attention. Moreover, the transformation of its solicitation from ancient to modern times is proof of the continuing role of natural products in health and wellness. You can bet that *N. sativa* will continue to be an energetic constituent of nutrition and medicine as long as investigation and development efforts invest in it that will yield even more beneficial outputs.

A comprehensive review of *N. sativa* , also known as black seed, casting its scope for novel product development, nutritional assistance, and medicinal usages, is the goal of this review. Functional food, traditional medication, and nutraceutical advancement are certain of its other possible solicitations. Besides existing concerns such as bioavailability and safety, the evaluation designates where medicine development and biotechnology can employ additional investigation and invention in the future.

## 2 Botanical Description of Black Seed

Black seed and black cumin are substitutes for one and the same plant species, Narcissus sativa of the Ranunculaceae family. According to Ahmad et al. (2021), the Latin “niger” or “black” is an explanation of the color of the seeds of the Nigella genus. The minor annual flowering herb grows up to 20–30 cm high and has subtle, thread‐like leaves that occur interchangeably (Shafodino et al. 2022). The distinctive Ranunculaceae flower shape consists of five to 10 small petals and a pale blue or whitish tint (Hannan et al. 2021). The massive, inflated capsule, which is the fruit of *N. sativa* , comprises a number of small, triangular seeds that occupy each of the three to seven amalgamated follicles (Srinivasan 2018). The kernel of the plant is the most vigorous constituent, and it is crowded with phytochemicals such as thymoquinone and alkaloids, and it has a bitter taste and a pungent scent (Ramadan 2021). North Africa, Southwest Asia, and parts of the Mediterranean are the natural environments of *N. sativa* . Southern Europe, the Middle East, and India have all been cultivating the plant for centuries due to its frequent culinary, medicinal, and beauty applications (Mohammed et al. 2016).

The plant grows best in temperate semi‐arid areas with typical yearly rainfall and freely draining sludge (Ramadan 2007). The plant flourishes best in lands rich in organic matter with a pH fluctuating from 6.5 to 7.5. In tropical and subtropical regions, it is generally sown in late autumn and harvested as a winter yield (Benazzouz‐Smail et al. 2023). In temperate regions, the seeds are sown directly into the soil at a depth of 1–2 cm during spring (Dalli et al. 2021). Being quite drought‐resistant and requiring a minimum of watering, it can thrive well in less water‐endowed areas, too (Datta et al. 2012). It is through manual harvesting that most farm cultivation around the globe takes place, whereby seeds are hand‐picked out of capsules so fresh that they may still have capillariums intact. Nonetheless, mass production adopted modern mechanical techniques, mainly in the Egyptian and Turkish commercial farms, besides the Indian mass ones (Michel et al. 2011).

One of the challenges with black seed production is susceptibility to weeds, pests, and fungal infections during germination and growth phases (Dinagaran et al. 2016). To ensure greater crop yield and conservation of the phytochemical properties of the seeds, organic fertilizers and integrated pest control measures are increasingly employed (Zribi et al. 2014). The practice of cultivation has been in antiquity in both the nation and commerce. Islamic wisdom confers a strong importance to black seed, prevalently mentioned to as the “seed of blessing” in the circumstance of prophetic medication (Hossain et al. 2021). Apart from being rummage‐sale items such as foods and medications, the seeds have long been employed in the circumstance of Ayurvedic as well as Unani medication (Sharma et al. 2011).

Pharmaceutical and cosmetic trades are among the most profitable sectors for the commercial usage of black seed oil. Demand for *N. sativa* has been cumulative internationally because of its cumulative status as a therapeutic product and functional food constituent (Adebayo‐Tayo et al. 2021). Coping with this accumulative demand will include sustained efforts to improve production procedures and increase territorial reach (Qayyum et al. 2020). In short, *N. sativa* 's plant and agronomic physiognomies demonstrate its flexibility and tenacity, which make it an extremely prospective crop in maximum zones. Its far‐reaching phytochemical profiles and ancient history typically authenticate its image of being universally applicable with numerous solicitations from industry to nutrition and wellbeing.

## 3 Nutritional Profile of Black Seed

**FIGURE 1:**
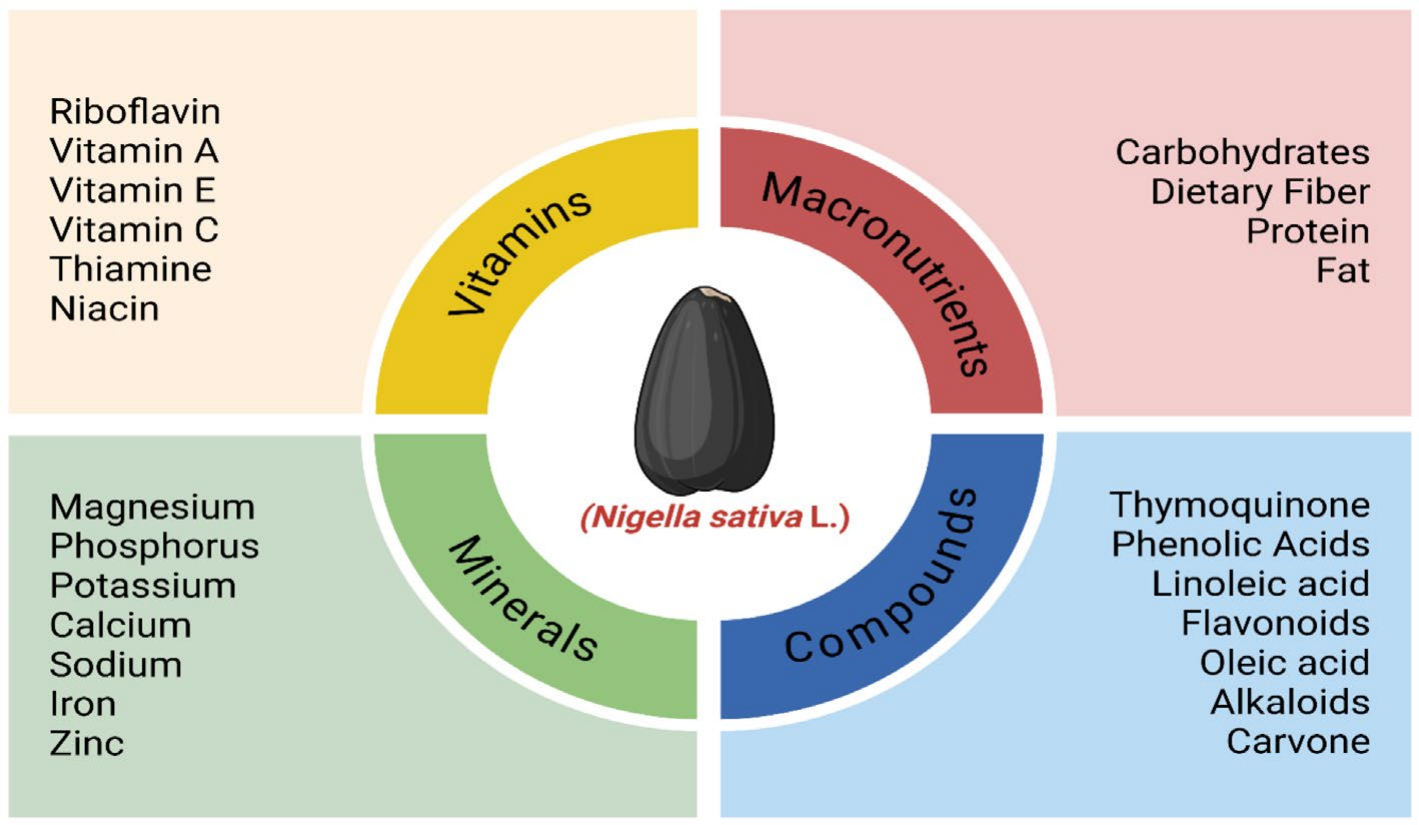
Composition of black seed.

Black seed comprises 20%–25% protein by weight and is an outstanding source of amino acids, predominantly valuable categories such as lysine and methionine (Ahmad et al. 2021). *N. sativa* is obligatory for cellular development and repair due to its proteins subsidizing enzymatic and structural roles in various biological procedures (Shafodino et al. 2022). Oleic acid (omega‐9) and linoleic acid (omega‐6) are two of the main fixed oils, constituting 30%–40% of seed weight (Ramadan 2021). Mohammed et al. (2016) have described that unsaturated fatty acids, predominantly oleic and linoleic acids, can assist in preserving heart health by decreasing cholesterol and inflammation levels (Liang et al. 2023). Apart from palmitic acid and other saturated fats, the kernel also has other ingredients that make it more thermally stable and long‐lasting on the shelf (Figure 1) (Srinivasan 2018).

30%–40% of the carbs from the dietary fiber of *N. sativa* kernels are in the form of carbohydrates. As a prebiotic, it endorses preservation of a well‐functioning digestive system through boosting regular bowel movements (Hannan et al. 2021). Black seed assists in diabetes therapy because of its high content of iron and fiber. Fiber content is also helpful in glycemic control (Dinagaran et al. 2016). Rich in calcium, magnesium, zinc, iron, and potassium, it is essential for upholding electrolyte balance, enzymatic activity, and bone wellbeing (Cheikh‐Rouhou et al. 2007). Magnesium sustains neuromuscular function; zinc and iron improve immunological role and oxygen transport, correspondingly (Datta et al. 2012). Vitamins such as thiamine (B1), riboflavin (B2), niacin (B3), and vitamin E, which is also called tocopherol, are rich in black seed. These serve as cofactors and antioxidants for metabolism (Benazzouz‐Smail et al. 2023). Explicitly, vitamin E enhances the skin's condition and defends cells from oxidative stress (Dalli et al. 2021).

## 4 Bioactive Compounds of Black Seed

### 4.1 Thymoquinone (TQ)

The main bioactive constituent, thymoquinone, is present at 30%–48% concentration in the essential oil composition of the plant. TQ has been recognized to have potent anti‐inflammatory, anticancer, antimicrobial, and antioxidant effects that make it an area of pharmacological study (Ahmad et al. 2021). Because of this property, TQ is also utilized as a management for chronic diseases, such as cancer and arthritis. It exerts its scavenging of free radicals and impacts the inflammatory pathway (Shafodino et al. 2022).

### 4.2 Alkaloids

Several alkaloids include nigellicine and nigellidine. The chemicals act through the pathways of neurotransmitters to show analgesic and anti‐inflammatory activities (Hannan et al. 2021). The presence of alkaloids shows antibacterial activity, thus stopping diseases from becoming resistant to various drugs (Srinivasan 2018).

### 4.3 Saponins

Another important category of phytochemicals present in black seeds, saponins, enhances immunological activity and reduces the intestinal absorption of cholesterol (Dinagaran et al. 2016). Additionally, these constituents have anticancer properties by activating cell death in cancerous tumors (Dajani et al. 2016).

### 4.4 Fixed Oils

Fatty acids are abundant in the fixed oils of kernels of *N. sativa* . These typically contain the fatty acids linoleic, oleic, and palmitic. These traits are accountable for the therapeutic advantage of *N. sativa* . The antioxidant and anti‐inflammatory properties of the oils defend cells from free radical damage and assist in decreasing inflammation related to chronic health conditions (Mohammed et al. 2016). Cosmetic products that integrate these oils have been shown to promote skin hydration and healing (Bourgou et al. 2012).

### 4.5 Phenolic Compounds

The antioxidant prospective of the plant is additionally improved by the great concentration of phenolic acids found in *N. sativa* , such as gallic acid and caffeic acid. Adebayo‐Tayo et al. (2021) described that these compounds hinder cell constituents from oxidative impairment by hindering lipid peroxidation.

### 4.6 Volatile Oils

The unique scent and medicinal properties of black seed are attributed to its volatile oil fraction, which comprises complexes such as p‐cymene, carvacrol, and thymoquinone (Hossain et al. 2021). These oils possess antibacterial properties that make them effective against a comprehensive variety of bacterial and fungal illnesses (Table 1) (Saleh et al. 2018). Table 1 designates that *N. sativa* is a pointer species for the nutritional and therapeutic qualities, and it has macro‐ and micronutrients and bioactive substances. The major phytochemicals like thymoquinone, alkaloids, saponins, and fixed oils having diverse pharmacological values depict the importance of functional foods and medicinal plants. Antibacterial, anti‐inflammatory, and antioxidant effects are just a few among numerous health benefits demonstrated by the combination of all of these features.

**TABLE 1:** Nutritional components, bioactive compounds, and benefits of black seed.

Component	Category	Benefits	References
Proteins	Macronutrient	Essential for muscle repair, immune function, and enzyme production	Singletary (2022)
Carbohydrates	Macronutrient	It provides energy and helps maintain blood sugar levels	Askari et al. (2019)
Dietary fiber	Macronutrient	It aids digestion, helps control blood sugar levels, and promotes gut health	Nikolić et al. (2020)
Fats (Lipids)	Macronutrient	It provides essential fatty acids like omega‐3 and omega‐6, contributing to cardiovascular health	Rahim et al. (2022)
Omega‐3 fatty acids	Fatty acids	It supports heart health, reduces inflammation, and enhances brain function	Alasalvar et al. (2021)
Omega‐6 fatty acids	Fatty acids	Essential for skin health, hormone regulation, and immune function	Alasalvar et al. (2021)
Thymoquinone	Phytochemical	It possesses anti‐inflammatory, antioxidant, and anticancer properties	Hannan et al. (2021)
Alkaloids	Phytochemical	Known for antimicrobial, analgesic, and anti‐inflammatory effects	Singletary (2022)
Saponins	Phytochemical	It helps in lowering cholesterol and acts as an antioxidant	Nikolić et al. (2020)
Fixed oils (black seed oil)	Bioactive compounds	It is high in essential fatty acids, supports skin health, and boosts immune function	Rahim et al. (2022)
Tocopherols (Vitamin E)	Micronutrient	An antioxidant that helps protect cells from damage, supports skin health, and boosts immunity	Albakry et al. (2022)
Vitamin C	Micronutrient	It enhances immune function, acts as an antioxidant, and aids collagen production	Alasalvar et al. (2021)
Iron	Mineral	It is essential for oxygen transport in the blood and energy production	Hannan et al. (2021)
Calcium	Mineral	Essential for bone health, nerve function, and muscle contraction	Hannan et al. (2021)
Magnesium	Mineral	It supports muscle and nerve function, regulates blood sugar levels, and promotes healthy bones	Rahim et al. (2022)
Zinc	Mineral	Necessary for immune function, protein synthesis, and cell division	Singletary (2022)
Potassium	Mineral	Regulates fluid balance and supports heart and muscle function	Singletary (2022)
Phytosterols	Bioactive compounds	Help reduce cholesterol levels and support heart health	Alasalvar et al. (2021)
Flavonoids	Bioactive compounds	Antioxidant and anti‐inflammatory effects protect against oxidative stress and chronic diseases	Hannan et al. (2021)
Sesquiterpene lactones	Bioactive compounds	It exhibits anti‐inflammatory and antimicrobial properties	Hannan et al. (2021)
Folate (Vitamin B9)	Micronutrient	Crucial for DNA synthesis, cell division, and red blood cell formation	Nikolić et al. (2020)
Phytochemicals (e.g., carotenoids)	Phytochemical	It acts as an antioxidant and supports vision, skin health, and immune function	Alasalvar et al. (2021)

## 5 Medicinal Applications of Black Seed

### 5.1 Antioxidant and Anti‐Inflammatory Properties

**FIGURE 2:**
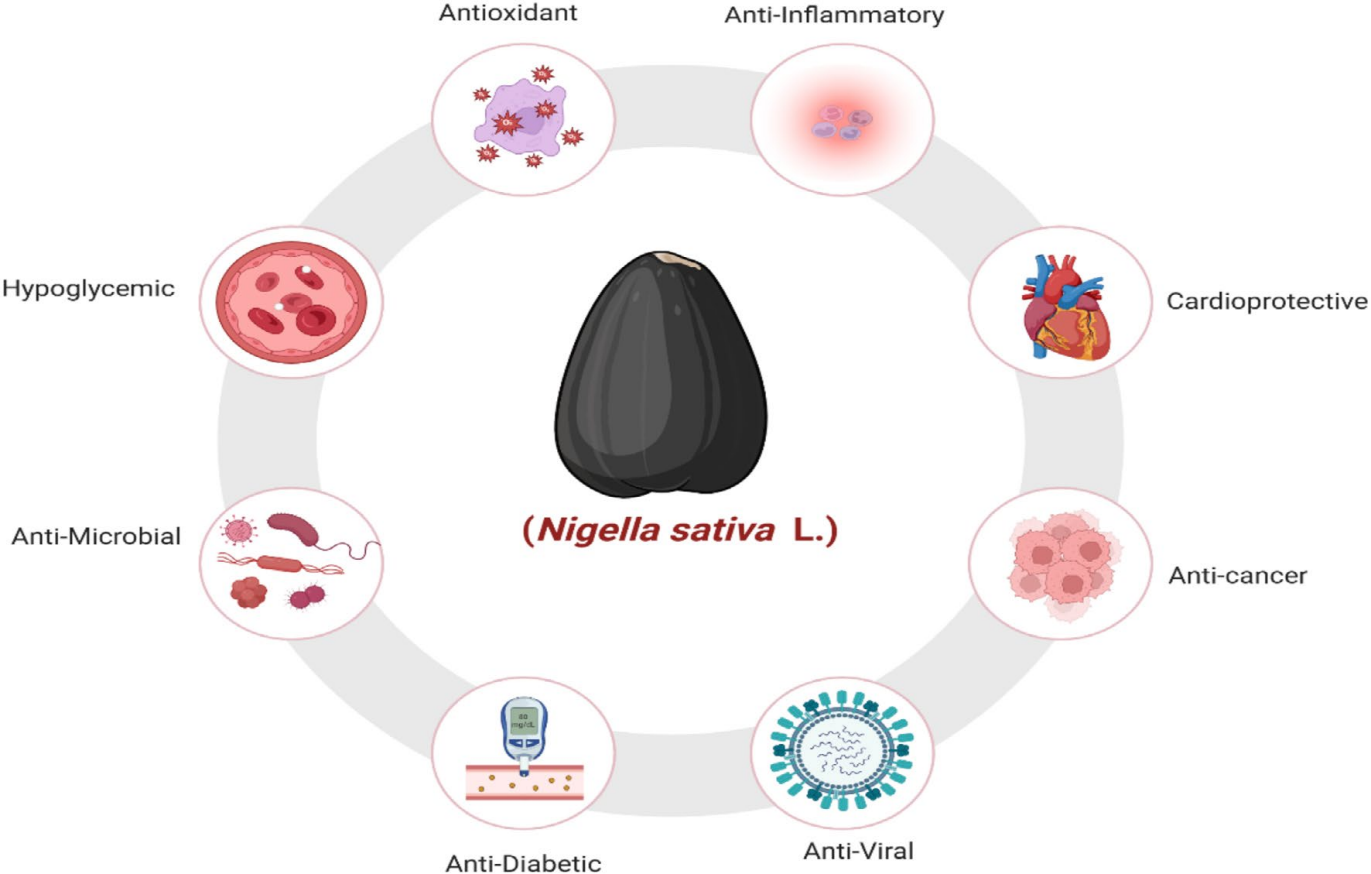
Health potential benefits of black seed.

*Nigella sativa*, also denoted as black seed, owns several medicinal properties and is of particular attention due to its antioxidant and anti‐inflammatory actions (ALRashdi et al. 2024). Thymoquinone (TQ), alkaloids, fixed oils, and other bioactive compounds are present in the kernels, and that is why they own these properties. Black seed is a much‐desired natural medicine in conventional and complementary medication because of the widespread studies that have demonstrated its therapeutic effectiveness in reducing oxidative stress and in the treatment of diverse inflammatory diseases. Investigations on the antioxidant activity of *N. sativa* have exposed encouraging results, as it is a vital factor in combating oxidative stress, an important causative factor for cancer, cardiovascular disease, and neurological disorders, to reference a few (Figure 2) (Ahmad et al. 2021).

Thymoquinone, flavonoids, and phenolic complexes are the main antioxidants in black seed. Bourgou et al. (2012) described that these composites cooperate to constrain lipid peroxidation, scavenge free radicals, and improve the antioxidant procedures of the body. Thymoquinone is the major ingredient in black seed oil with strong antioxidant activity. Studies have indicated that TQ can scavenge free radicals and shield cells against the toxic influence of oxidative stress (Mohammed et al. 2016). Further, catalase and SOD are antioxidant enzymes that shield cells against oxidative harm; TQ enhances their activity (Kazemi 2014). Moreover, various organs, such as the liver and brain, have also shown that black seed oil decreases oxidative stress. It may be useful to avert and treat disorders associated with oxidative stress (Dajani et al. 2016). Other than this, numerous studies have indicated the coordination of phenolic chemicals responsible for the increment of antioxidant activity. It also shows that it possesses a high content of phenolic acids, of which gallic acid acts mainly to scavenge oxidative free radicals in destroying dangerous cellular damage (Dinagaran et al. 2016).

Several studies also showed that research on various methods of black seed oil extraction revealed that antioxidant properties varied with the different extraction methods. So, method optimization is an important part of maintaining bioactive ingredients (Mohammed et al. 2016). Equally as well recognized are its anti‐inflammatory properties that would provide support for using the seed in the therapy of rheumatoid arthritis, asthma, and other chronic inflammatory conditions. While most of the observed inflammation modulation is attributed to thymoquinone, other compound classes, including alkaloids and saponins, are also crucial in reducing inflammation (Amin and Hosseinzadeh 2016). Thymoquinone functions through several mechanisms to control inflammation. It was documented to reduce the production of cytokines such as tumor necrosis factor‐alpha (TNF‐α), IL‐6, and IL‐1β that promote inflammation by inhibiting nuclear factor kappa‐light‐chain‐enhancer of activated B cells (NF‐κB) activation, a fundamental transcription factor in inflammation (Bordoni et al. 2019). COX‐2 is another enzyme necessary for inflammation. Investigations carried out by Rashwan et al. (2023) have designated that TQ can downregulate the expression of this explicit enzyme. Because of these mechanisms, black seed may be an appreciated therapeutic agent in contradiction to inflammatory diseases such as arthritis and inflammatory bowel syndrome.

Animal‐based investigations have exposed that *N. sativa* owns potent anti‐inflammatory activity. For illustration, an inflamed disorder known as paw edema was lessened in rats through black seed oil by averting inflammatory mediators (Gholamnezhad et al. 2015). Clinical trials have demonstrated that black seed treatment has been shown to reduce human inflammatory markers and thus develops a desirable interference policy for inflammatory diseases (Rashwan et al. 2023). Besides thymoquinone, other bioactive composites found in black seed, such as nigellidine and nigellicine, add to its anti‐inflammatory activity. By affecting frequent inflammatory trails, these substances have exposed probable diseases such as asthma and chronic obstructive pulmonary disease (COPD) (Ojueromi et al. 2022). Prostaglandins and nitric oxide (NO), dual key inflammation intermediaries, may be congested by the fixed oils of *N. sativa* , as designated in studies (Kazemi 2014).

The antioxidant and anti‐inflammatory properties of *N. sativa* validate it as a prospective drug for the management of a variety of disorders associated with oxidative stress and inflammation. For example, studies have revealed that *N. sativa* can decrease particular manifestations of asthma, a disease categorized by oxidative stress and airway inflammation. Animal investigations on asthma have designated that black seed oil improves airway inflammation and reduces free radical scavenging (Gholamnezhad et al. 2015). Additionally, diseases in which oxidative stress is a key driving force behind their development, e.g., eczema and psoriasis, can be enhanced by the joint action of antioxidant and anti‐inflammatory properties. Topical treatment with *N. sativa* oil has shown important effectiveness as a therapeutic agent to decrease redness, inflammation, and irritation seen in these disorders (Bordoni et al. 2019). Because *N. sativa* controls the immune system, this will improve its efficiency in fighting chronic inflammatory illnesses. Numerous studies have shown that *N. sativa* extract improves a healthier immune system by enhancing the activity of anti‐inflammatory cytokines while decreasing pro‐inflammatory cytokine activity (Dajani et al. 2016).

Scientific examination has authenticated that black seed, or *N. sativa* , comprises pharmacological activity, making it an influential antioxidant and anti‐inflammatory. Its bioactive composites, such as thymoquinone, alkaloids, and phenolic acids, comprise anti‐inflammatory and free radical scavenging activities. Black seed is thus a natural remedy for inflammatory situations and oxidative stress‐related diseases. More investigations are wanted to have a full understanding of its action mechanisms and prospective therapeutic solicitations, mainly in clinical settings.

### 5.2 Antimicrobial and Antiviral Effects

For quite a while, traditional medication has taken benefit of the assistance of *N. sativa* , usually denoted as black seed. The antiviral and antibacterial characteristics of black seed have concerned much consideration because of its prospective natural solicitation in the cure of different circumstances (Table 2). The pharmacological activity of black seed in contradiction of fungal, viral, and bacterial infections is due to bioactive composites contained within it, such as thymoquinone, alkaloids, and vital oils. The molecular mechanisms and potential solicitations of *N. sativa* , with antibacterial and antiviral possessions, are deliberated in this segment. Many studies have been done in order to evaluate *N. sativa's* antibacterial properties, which prove potent against a variety of diseases that also include bacteria, fungi, and parasites. The main bioactive compound responsible for these actions is thymoquinone (TQ); however, melanthin and nigellidine have a bactericidal effect (Forouzanfar et al. 2014).

**TABLE 2:** Health benefits of black seed with case studies.

Activity	Disease	Case study	References
Antioxidant and anti‐inflammatory	Chronic inflammation	Bordoni et al. (2019) demonstrated that *Nigella sativa* oil reduced inflammation and oxidative stress in human pre‐adipocytes, highlighting its potential for treating chronic inflammation	Bordoni et al. (2019)
	Obesity‐related Inflammation	Ojueromi et al. (2022) found that *Nigella sativa* oil showed significant anti‐inflammatory effects in obese individuals, potentially supporting its use in obesity‐related inflammatory diseases	Ojueromi et al. (2022)
Antimicrobial and antiviral	Respiratory infections	Salem and Hossain (2000) found that black seed oil protected against murine cytomegalovirus infection, suggesting antiviral potential for respiratory viral infections	Salem and Hossain (2000)
	COVID‐19	Islam et al. (2021) suggested that *Nigella sativa* 's thymoquinone could prevent and treat COVID‐19 through its antiviral properties	Islam et al. (2021)
Anticancer	Breast cancer	Shafiq et al. (2014) demonstrated that *Nigella sativa* exhibited anticancer properties against breast cancer cells, suggesting its potential use as an adjunct in cancer therapy	Shafiq et al. (2014)
	Colorectal cancer	Majdalawieh and Fayyad (2016) observed that thymoquinone, a key compound in *Nigella sativa* , inhibited cell proliferation and induced apoptosis in colorectal cancer cells	Majdalawieh and Fayyad (2016)
Hypoglycemic and antidiabetic activity	Type‐2 diabetes	Hamdan et al. (2019) found that *Nigella sativa* supplementation significantly reduced fasting blood glucose levels in patients with type 2 diabetes	Hamdan et al. (2019)
	Insulin resistance	Bamosa (2015) reviewed studies showing that *Nigella sativa* 's thymoquinone exerted hypoglycemic effects and improved insulin sensitivity in diabetic patients	Bamosa (2015)
Cardioprotective benefits	Hypertension	Rashwan et al. (2023) found that *Nigella sativa* significantly lowered blood pressure in hypertensive patients, indicating its potential as a natural treatment for cardiovascular diseases	Rashwan et al. (2023)
	Atherosclerosis	Hannan et al. (2021) reported that *Nigella sativa* 's antioxidant properties helped reduce oxidative stress and inflammation, key contributors to atherosclerosis	Hannan et al. (2021)

The majority of research has proven black seed oil and extracts to possess antibacterial properties against Gram‐positive and Gram‐negative bacteria. *Salmonella typhimurium* , *Pseudomonas aeruginosa* , *Staphylococcus aureus* , and *Escherichia coli* are some of the bacteria most commonly affected by black seed. Black seed attacks the cell membranes of bacteria and prohibits the formation of the bacteria's cell walls; further, it also obstructs the synthesis of proteins in bacteria (Abdallah 2017; Ahmad et al. 2021). Part of the outstanding property of thymoquinone is its capability to inhibit the growth of bacteria through modification of bacterial cell membrane integrity, thereby forcing vital cellular elements to leak out, ultimately leading to death (Basurra et al. 2021).

Moreover, combined with conventional antibiotics, black seed oil has exhibited synergistic advantages in enhancing their efficacy and minimizing the emergence of antibiotic resistance (Forouzanfar et al. 2014). Also, black seeds were demonstrated to be extremely antifungal. Numerous fungal infections, such as *Candida albicans* , *Aspergillus flavus*, and *Trichophyton rubrum*, were treated proficiently by it. These fungi are responsible for numerous varieties of infections, fluctuating from superficial skin infections to Spartan systemic diseases. The antifungal action of *N. sativa* is supposed to be the consequence of its capability to constrain the growth of fungi and sporulation. This is likely done by interfering with cell membranes and overwhelming enzyme activity that is accountable for the amalgamation of cell wall constituents, as described by Basurra et al. (2021). Black seed oil, as per investigation, comprises fungicidal possessions that, when rummage‐sale in advanced concentrations, kill fungal cells and also overwhelm their growth. An indication that validates the antifungal activity of black bean oil is its capability to hinder biofilm formation, a typical feature of fungal infections (Shamim Molla et al. 2019).

*Nigella sativa* acts alongside bacterial and fungal sicknesses, but it also has prospective action against parasitic sickness. For instance, numerous studies have designated that *N. sativa* substantially hinders the parasite *Plasmodium falciparum*, which causes malaria. The antiparasitic activity of thymoquinone in black seeds is supposed to be the consequence of its capacity to regulate the immune system and impede the metabolic activity of parasites (Abbas et al. 2024). Moreover, the anthelmintic properties of black seed have been well examined and utilized in treating parasitic diseases of the gastrointestinal tract (Yimer et al. 2019). The antiviral activity of *N. sativa* has of late been subject to a lot of scrutiny, particularly in the wake of augmented viral infections such as COVID‐19, the common cold, flu, and hepatitis. Investigations have identified some of the diverse mechanisms through which thymoquinone and other bioactive compounds present in black seed overwhelm viral replication and modulate host immune response. One of the primary mechanisms through which black seed fights viruses is by directly interacting with them, thereby preventing them from binding to and entering the host cells. Firstly, intended to wedge the entry, assembly, and release of freshly synthesized virions, thymoquinone now activates at numerous points of the viral replication cycle. Additionally, *N. sativa* is capable of modifying the host immune response and increasing interferon production, two major components of antiviral defense (Shamim Molla et al. 2019).

Black seed's antiviral activity has been investigated primarily in terms of its effectiveness against respiratory viruses, comprising coronaviruses and influenza. Investigations have exposed that *N. sativa* can constrain the attachment of the influenza virus to host cells, thus preventing the virus from replicating. Research has shown that thymoquinone overwhelms viral replication by preventing the viral RNA polymerase enzyme (Islam et al. 2021). Investigations designated that black seed can be exploited as an additional treatment for COVID‐19 when the epidemic broke out. Jassey et al. (2022) specified that thymoquinone predominantly represses SARS‐CoV‐2 replication by plummeting the inflammatory cytokine storm and delaying the entry of viruses. In addition, black seed oil can decrease the severity of viral infections because it has immunomodulatory effects that are obliging in controlling the immune system (Basurra et al. 2021).

Black seed also owns antiviral herpes simplex virus (HSV) and hepatitis virus (HBV) activity. Hepatitis B and C viruses cause chronic liver disease, and it has been recognized that *N. sativa* overwhelms their replication. Liu et al. (2025) recognized that the antiviral activity of thymoquinone includes the modification of the immune response and resolution of inflammation in the liver as a consequence of the growth retardation of the virus. In the case of herpes simplex virus (HSV), investigations have specified that black seed oil can decrease the viral load in the host immune system and hinder the virus directly from replicating, which accelerates the process of lesion healing (Abdallah 2017).

Due to its antiviral and antimicrobial properties, *N. sativa* is a potent natural treatment for various diseases. Of its bioactive compounds, thymoquinone is predominantly noted for its capability to fight parasites, germs, and viruses. As an antibacterial, black seed can possibly stem drug‐resistant diseases while at the same time increasing the efficiency of more traditional drugs. Most helpful in the treatment of respiratory viruses and long‐lasting viral diseases such as hepatitis, its antiviral effect underlines its medicinal value in up‐to‐date medicine. *N. sativa* is poised to become an outstanding natural therapy for infectious illness as more examination into its molecular mechanisms and medicinal applications is done.

### 5.3 Anticancer Activity

There have been numerous studies on the medical solicitations of *N. sativa* , frequently mentioned as black cumin or black seed, and certain of them have even linked it with cancer therapy. This versatile plant has a long history of usage in folk medicine for an array of disorders, such as infections and digestive glitches. Thymoquinone, a bioactive compound of *N. sativa* , has garnered a lot of consideration recently due to its prospective solicitation in the anticipation and treatment of cancer (Majdalawieh and Fayyad 2016). Exploring the mechanisms of therapy, clinical indication, and prospective applications of *N. sativa* , the review is focused on its anticancer properties. Alkaloids, saponins, flavonoids, and essential oils are all ingredients of *N. sativa* 's large phytochemical profile that lends it its therapeutic properties. Thymoquinone, the major bioactive compound, has been the focus of much consideration due to its high anticancer activities. Thymoquinone possesses a number of biological activities, such as distressing the immune system, decreasing inflammation, and defending cells against free radicals. Investigation from Khan et al. (2011) that thymoquinone overwhelms metastasis, promotes apoptosis, and inhibits cancer cell growth. This makes it an operative anticancer drug.

Thymoquinone, which targets some of the biochemical pathways involved in carcinogenesis, is supposed to be the major action through which it exerts its anticancer effects (Khalid et al. 2020). One mechanism has been the promotion of apoptosis in cancer cells as part of the main machinery involved in anticancer effects, mainly inducing caspases and modifications of expression levels in pro‐apoptotic and anti‐apoptotic proteins. Further impeding the proliferation of tumors is the ability of thymoquinone to obstruct angiogenesis—the process by which tumors physic their blood supply (Majdalawieh and Fayyad 2016). Thymoquinone also has anticancer effects through the modification of the expression of some signaling molecules and enzymes that are critical in the development of cancer. For instance, it inhibits NF‐κB, a transcription factor that has been implicated in inflammation and cancer development. NF‐κB blocks the synthesis of pro‐inflammatory cytokines, which are often overexpressed in malignant tissues, thus reducing their synthesis. Moreover, thymoquinone was found to inhibit the activities of some important enzymes linked with the processes of invasion and metastasis of cancer cells, which include matrix metalloproteinases (MMPs) and cyclooxygenase‐2 (COX‐2) (Mollazadeh et al. 2017).

Numerous studies have designated that *N. sativa* and its active ingredients own anticancer activity, both in vivo and in vitro. Cell culture studies have designated that thymoquinone constrains the growth of numerous cancer cells, such as those present in the liver, breast, lungs, colon, and prostate. For example, thymoquinone has been described to decrease cell proliferation of MCF‐7 breast cancer cells, improve apoptosis, and constrain cell cycle progression through the G1 phase (Khurshid et al. 2020). Indication designates that extracts of *N. sativa* can overwhelm cancer cell growth and metastasis in animal prototypes.

**FIGURE 3:**
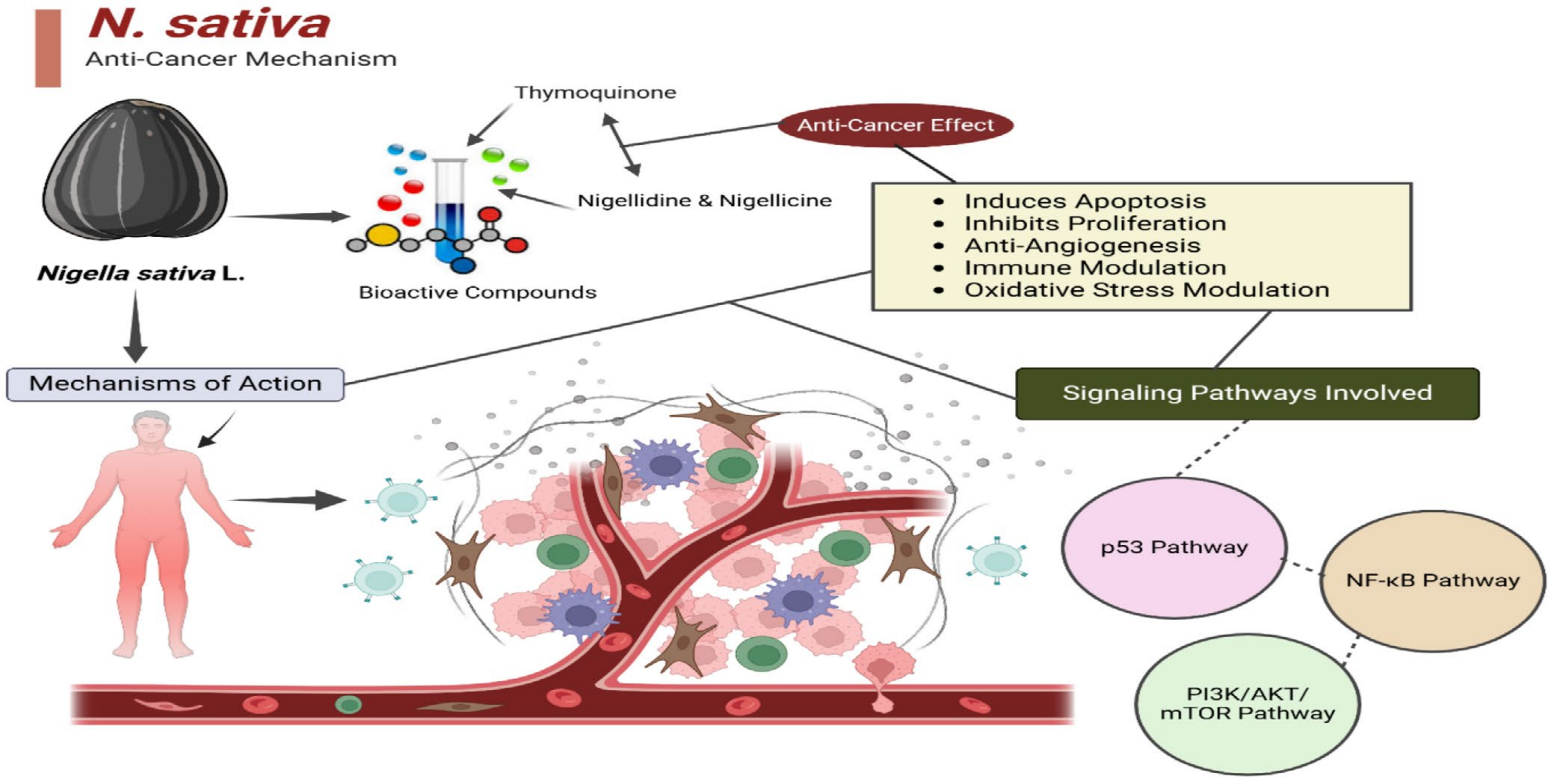
Anticancer mechanism of action of black seed.

Black seed extract can possibly constrain tumor development in breast cancer mice through improved immune responses and downregulation of tumorigenic markers' expression, as per Ait Mbarek et al. (2007). An additional possible solicitation of *N. sativa* in cancer therapy is as an adjuvant therapy, as it has been found to exhibit more effective anticancer activity when rummage‐saled in amalgamation with standard chemotherapy drugs (Agbaria et al. 2015). There is limited human clinical indication of the anticancer activity of *N. sativa* , although preclinical investigation has been promising. However, some encouraging consequences have been stated from clinical trials, chiefly for breast and colorectal cancers. In a clinical trial of *N. sativa* 's possessions on breast cancer, for instance, Esmaeli et al. (2024) described that when administered in amalgamation with conventional actions, it reduced tumor size, resulting in improved patient consequences. The immunomodulatory possessions of *N. sativa* , which prompt the body to abolish cancer cells, may also augment the effectiveness of conventional cancer treatments such as radiation and chemotherapy (Figure 3) (Esmaeli et al. 2024).

*Nigella sativa* owns prospective anticancer activity, but a few obstacles are yet to be overcome before the plant can be rummage‐sale clinically. The furthermost critical is the requirement for constancy in dosage regimens and formulations. Numerous studies have exploited several forms of *N. sativa* extracts, and controversy exists concerning the optimal dosage or direction technique for anticancer effectiveness. Additional apprehensions over the bioavailability of bioactive substances such as thymoquinone are due to the jeopardy that these may be quickly metabolized or cleared before attaining therapeutic levels in the body (Hamadain et al. 2019). There is a requirement for additional investigations to establish its long‐term safety in cancer patients, inaugurate standardized treatment regimens, and improve its therapeutic prospective. The destiny of cancer therapy lies in the hands of *N. sativa* , due to its massive pharmacological activities and lower toxicity.

### 5.4 Hypoglycemic and Antidiabetic Effects

**FIGURE 4:**
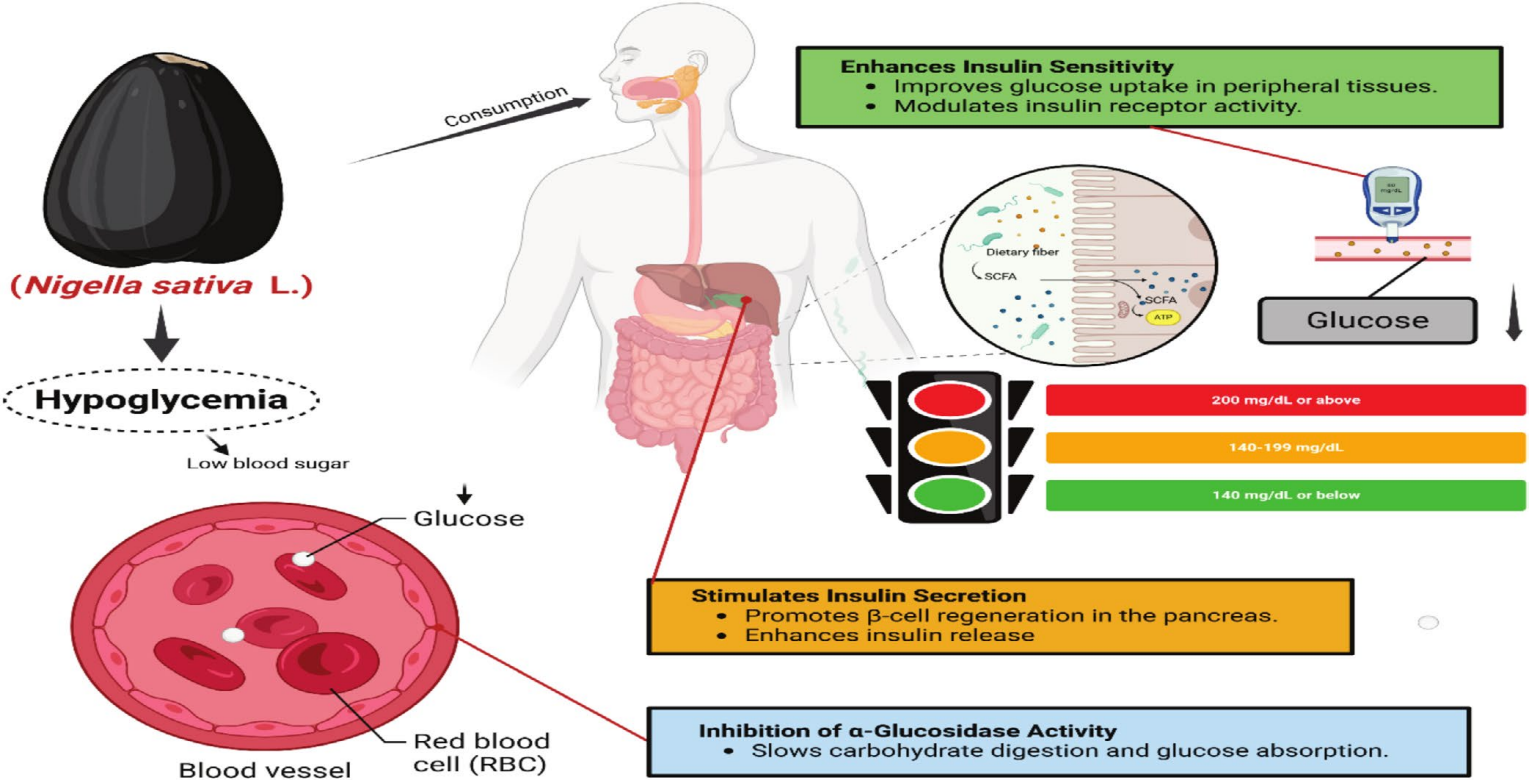
Antidiabetic mechanism of action of black seed.

*Nigella sativa* finds a diversity of solicitations in traditional medicine across numerous countries because black seed, black cumin, and other terms have numerous therapeutic properties. *N. sativa* is of substantial attention with regard to the hypoglycemic and antidiabetic accomplishments due to its other therapeutic compensations (Srinivasan 2018; Maideen 2021). The antidiabetic activity of black seed, chiefly accredited to the active compound thymoquinone, renders it a very stimulating plant for the control of diabetes mellitus. Explaining its mechanisms of action, experimental results, and possible therapeutic usage, this article is an inclusive examination of *N. sativa* hypoglycemic and antidiabetic actions. *N. sativa* seeds have therapeutic properties because they comprise numerous bioactive compounds. Some of these compounds include essential oils, saponins, alkaloids, and flavonoids. Most of the medicinal attributes of *N. sativa* , such as its antidiabetic and hypoglycemic effects, have been ascribed to thymoquinone (TQ), the best‐studied active constituent of the plant (Figure 4). Thymoquinone's ability to treat diabetes is bolstered by its range of biological activities, such as immunomodulatory, anti‐inflammatory, and antioxidant effects (Maideen 2021).

A number of composites, such as thymoquinone, carvacrol, and t‐anethole, have revealed promise for improving glucose metabolism and insulin sensitivity. These composites lend weight to the plant's antidiabetic influences (Srinivasan 2018). The hypoglycemic and antidiabetic properties of *N. sativa* are attributed to a diversity of paths that impact glucose metabolism. One of the most important avenues is the regulation of insulin secretion and sensitivity. Studies have shown that *N. sativa* triggers pancreatic β‐cells to release more insulin, enhancing tissue glucose uptake. Benhaddou‐Andaloussi et al. (2011) discovered that *N. sativa* reduces blood glucose levels through the enhancement of GLUT4 expression in muscle cells, enhancing glucose uptake. The AMP‐activated protein kinase (AMPK) pathway is a vital energy‐balancing regulator of the cell; thymoquinone, for instance, was shown to activate this pathway. AMPK activation enhances insulin sensitivity, increases glucose uptake in peripheral tissues, and decreases gluconeogenesis in the liver, according to Benhaddou‐Andaloussi et al. (2011) and Shaukat et al. (2023).

Mathur et al. (2011) assert that *N. sativa* has antioxidant activity that counteracts oxidative stress, a major contributor to insulin resistance and β‐cell dysfunction in diabetes. The antidiabetic activity of *N. sativa* has been shown in a number of in vitro and in vivo preclinical studies. Experimental evidence indicates that *N. sativa* extracts have the potential to improve lipid profiles, regulate pancreatic functions, and reduce blood glucose levels drastically in mice. For instance, the 2015 study by Heshmati and Namazi examined the impact of *N. sativa* on metabolic markers in diabetic rats. The findings have shown that the herb improves one's insulin sensitivity and significantly lowers blood glucose levels. Likewise, Alhodieb (2022) showed that black seed extract yields noteworthy hypoglycemic effects by significantly reducing fasting blood glucose levels in rats (Alhodieb 2022). These results have been reinforced by in vitro studies using cell cultures. It has been proven that thymoquinone, the major bioactive compound of N. sativa, reduces glucose levels in cultured hepatocytes by inhibiting gluconeogenesis (Bamosa 2015).

Additionally, studies on human pancreatic cells have indicated that extracts from can protect β‐cells from oxidative damage, which is one of the common presentations of diabetes, and augment insulin secretion (Desai et al. 2015). Although most studies on the antidiabetic properties have been conducted using animal models, several clinical trials have also explored its efficacy in treating diabetes in humans.

Hamdan et al. (2019), in a systematic review, reported that it has positive effects on patients with type 2 diabetes. For example, extracts given as supplements to people with diabetes reduced blood glucose values significantly, increased insulin sensitivity, and improved glycemic management in clinical studies (Hamdan et al. 2019). This particular clinical trial by Maideen (2021) assessed the antidiabetic activity of black seed in humans; findings of this study indicated significant declines in fasting blood glucose levels and HbA1c, which measures long‐term glucose management after daily supplementation with black seed. Black seed can potentially reduce the risks associated with cardiovascular diseases secondary to diabetes, according to the lipid profile improvement obtained from the study (Maideen 2021).

Notwithstanding these promising results, little clinical data is validating the long‐term safety and efficacy of in diabetes management, and larger human studies are required to confirm these benefits and optimize dosing. It is typically considered safe when taken in moderation as a supplement or as part of a diet. However, side effects such as bloating and gas could result from misuse. Investigations have demonstrated that the plant extract is not damaging for laboratory creatures to take, even in greater dosages (Bensiameur‐Touati et al. 2017). It is, therefore, important to be on the lookout for any conceivable connections with other medications, particularly those used to regulate blood glucose, just like with any therapeutic mediator, in order to prevent hypoglycemia. An increasing volume of data supports the hypoglycemic and antidiabetic properties of black seed, especially that of its main ingredient, thymoquinone. Due to its impact on insulin sensitivity, oxidative stress, and glucose metabolism, *N. sativa* has been found to be a potentially valuable natural product for diabetes. Further investigation is necessary to find the full range of therapeutic assistance of *N. sativa* in diabetic patients, as well as consistent doses and long‐term safety. Although positive outcomes from preclinical and clinical trials suggest much potential, these fields are underdeveloped. *N. sativa* is prospective as an add‐on or supplement to diabetes treatment due to the diversity of pharmacological properties coupled with its profile of fewer risks (Maideen et al. 2024).

### 5.5 Cardioprotective Activity

Black seed, or *N. sativa*, is one of the composites that has gathered a lot of consideration with respect to medical solicitation. Though black seed has discovered numerous applications, the cardiovascular disorders that it is capable of resisting have fascinated experts with respect to its cardioprotective activities. Zeroing in on the active elements of the plant, mainly thymoquinone, and their mechanisms in keeping cardiac function by anti‐inflammatory, antioxidant, and other mechanisms, this article observes the cardioprotective potential of *N. sativa* . Alkaloids, flavonoids, saponins, essential oils, fatty acids, and vital oils are some of the bioactive composites found in *N. sativa* . As per Hannan et al. (2021), the cardiovascular benefits of black seed are mainly attributed to its most deliberate active component, thymoquinone (TQ).

TQ has a lot of noteworthy pharmacological properties, comprising antioxidant, anti‐arrhythmic, and anti‐inflammatory properties, that contribute to the management and prevention of cardiovascular disease (Shabana et al. 2013). Thymoquinone is not the sole compound in the herb with valuable effects on cardiovascular functions; carvacrol and t‐anethole are two additional ones (Srinivasan 2018). *Nigella sativa* enhances endothelial function and alleviates oxidative stress, inflammation, and dyslipidemia, among other cardiovascular health factors that are addressed by *N. sativa* through its cardioprotective action. To a large extent, *N. sativa* 's cardioprotection can be best illustrated by its high antioxidant activity. The progression of cardiovascular diseases such as atherosclerosis, myocardial infarction, and heart failure is significantly influenced by oxidative stress due to the loss of equilibrium between free radicals and antioxidants. The major bioactive compound of *N. sativa*, thymoquinone, lowers cardiovascular damage through scavenging of free radicals and inhibition of lipid peroxidation (Hannan et al. 2021).

One of the primary concerns in myocardial infarction patients is the preservation of the cells in the heart, and its antioxidant ability is a tremendous help toward that end. Chronic inflammation also plays a significant role in cardiovascular diseases. It prevents the synthesis of several pro‐inflammatory cytokines, such as TNF‐α, IL‐6, and IL‐1β, that are involved in the pathogenesis of cardiovascular diseases (Shabana et al. 2013). It has been shown that thymoquinone downregulates inflammation markers, reducing vascular inflammation and improving cardiovascular function. It prevents the impairment of endothelial function, a landmark step in the establishment of atherosclerosis‐required anti‐inflammatory action. One of the most important risk factors for cardiovascular diseases is dyslipidemia, which is characterized by high triglycerides, LDL (low‐density lipoprotein), and total cholesterol. Studies have shown that supplementing with it can improve lipid profiles by increasing HDL (high‐density lipoprotein) and decreasing triglycerides and total cholesterol (Shafiq et al. 2014). The jeopardy of atherosclerosis and associated cardiovascular trials is lessened because plaques in the arteries cannot form by dropping these atherogenic lipids. The vascular tone and blood flow are mostly maintained by the endothelium that lines all blood vessels. One of the main hallmarks of endothelial dysfunction, and a very early sign of cardiovascular disease, is lowered nitric oxide production, leading to lesser vasodilation. Previous studies had indicated that treatment with it resulted in improved vasodilation with an increase in NO levels and, thus, possible improvement in endothelial function. This widening of blood vessel walls assists in reducing blood pressure and keeps diseases such as high blood pressure and heart disease at bay, according to Ali et al. (2024).

Numerous in vivo investigations have established robust indications of the cardioprotective properties of *N. sativa* . To validate the case in point, Ghoreyshi et al. (2020) deliberate the function of *N. sativa* extract on ischemia–reperfusion‐induced oxidative stress and functional recovery in rat hearts. Ghoreyshi et al. (2020) and Ali et al. (2022) had gotten the consequence that hydroalcoholic extract of black seeds abridged oxidative stress meaningfully and enhanced cardiac function, representing that it may serve as rehabilitation for myocardial infarction and associated disorders. *N. sativa* also owns well‐established anti‐arrhythmic properties. Malihi et al. (2020), in their investigation involving isolated rat atria, established that thymoquinone would have the potential to constrain ouabain‐induced arrhythmias. This amount is rather useful since arrhythmias are a recurrent side effect in patients with cardiac pathology (Malihi et al. 2020). Even though most investigations on *N. sativa* 's cardioprotective properties have been done on animals, new clinical evidence designates that it might even have valuable influences on human cardiovascular well‐being.

A large‐scale investigation of *N. sativa* supplementation's effects on endothelial function in humans was accomplished by Ali et al. (2024). Endothelial function was also meaningfully enhanced by *N. sativa* , as designated by a greater flow‐mediated dilatation (FMD), or vascular health marker. With this in consideration, supplementation with black seeds can hypothetically decrease the risk of cardiovascular diseases, particularly among patients who are at greater risk of developing atherosclerosis and endothelial dysfunction (Ali et al. 2024). Clinical research has recognized that supplements of *N. sativa* may assist in regulating blood pressure and changing lipid profiles of hypertensive patients with hyperlipidemia (Shafiq et al. 2014). Altogether, these findings indicate that *N. sativa* could exert therapeutic effects against cardiovascular risk factors (Suleman et al. 2023).

Most individuals believe *N. sativa* is entirely safe to consume in moderate amounts as a food additive or meal supplement. The majority of the research on *N. sativa* supplementation has produced no significant negative effects. Anticoagulants and antihypertensive agents, among other drugs, would need to be monitored closely for any interaction as a result of the drug's potential impact on blood pressure as well as on platelet aggregation (Hussain and Hussain 2016). The cardioprotective action of *N. sativa* has been established by both animal and human studies. Thymoquinone, which is one of the active constituents of black seed, possesses anti‐inflammatory, antioxidant, lipid‐lowering, and anti‐arrhythmic activity, among numerous others. All things considered, *N. sativa* holds vast potential as a natural medication for preventing and treating cardiovascular disease. While more clinical trials are necessary to establish how best to dose *N. sativa* and keep it safe long‐term, it holds great promise as an adjunct treatment for heart health.

## 6 Role of Black Seed in Traditional Medicine

Black seed, or scientifically referred to as *N. sativa* , has been used by traditional medicine for a long time. The herb plays a number of roles and is greatly valued in many traditional systems of medicine, such as Ayurveda, Unani, and folk medicine. The herb's great value to both mainstream and complementary medicine lies in its well‐documented therapeutic properties. The cultural and historical background, traditional medicinal applications, and development of *N. sativa* from folk medicine to contemporary pharmacological studies are all discussed in this essay.

### 6.1 Black Seed in Ayurveda

Its origin lies in ancient India, where this world‐renowned medicinal practice was born. Nutrition, dietary adjustments, and natural treatments are all means by which *N. sativa* strives to restore the energy level in people. The herb's capacity to balance the Pitta, Kapha, and Vata doshas is well documented in Ayurveda. It is believed to benefit digestion, circulation, and mucus discharge due to its hot, strong, and bitter attributes (Srinivasan 2018). Ayurvedic treatments apply seeds for several disorders related to the skin, digestive, and respiratory tracts. For example, on account of its exploration and anti‐inflammatory properties, black seed is a drug usually prescribed to be taken in cases such as coughing, bronchitis, and asthma.

Moreover, it has also been applied to various cases of a disorder called indigestion. End. Black seed is often used in Ayurvedic medicine as oil, powder, or tea infusion and can help balance the body's processes naturally. Ayurvedic systems emphasize support in longevity and vigor. It helps maintain a well‐functioning digestive system while simultaneously augmenting immunity. This makes sense in accordance with the general belief of Ayurvedic views for maintaining body and mind balance. The Ayurveda medicine practice has continued to comprise the usage of black seed, while the new studies offer some evidence for its healing possessions (Majeed et al. 2021).

### 6.2 Black Seed in Unani Medicine

The medicinal attributes of *N. sativa* were initially noted in Greek medicine and later on refined in Islamic medicine; Unani medicine has also recognized such attributes. A sacred herb in Unani medicine, black seed (or “Kalonji” in its original India) has been said to possess curative qualities against many types of diseases. Unani medicine employs black seed as a drug for numerous illnesses, ranging from skin diseases, immune system disorders, gastrointestinal disturbances, and respiratory diseases. *N. sativa* is considered a rummage sale in Unani medicine because it supposedly contains “hot” and “dry” properties that balance the humors of the body, particularly when there is an excess of cold or phlegm. Arthritis, asthma, and gastrointestinal disorders are just a few of the numerous conditions that are treated with black seed by Unani practitioners. Topical uses of *N. sativa* oil have been shown to bring relief to skin disorders such as eczema and acne due to its antimicrobial and anti‐inflammatory properties (Dabeer et al. 2022).

This is deeply rooted in Unani medicine, given the importance the prophetic saying in Islamic tradition has placed on black seed: “Black seed is a cure for every disease except death.” The wide uses of this system today testify to the long history and deep cultural origins of black seed as a universal cure. The unremitting use of black pits in Unani rituals is buttressed empirically by modern scientific inquiry that has started to authorize many of these traditional resolutions (Usmani and Almoselhy 2023).

### 6.3 Folk Medicine and Cultural Significance

Apart from the official systems of Ayurveda and Unani, it has been extensively used in many folk medical traditions all over the world. In most Middle Eastern and North African civilizations, black seed is considered an integral component of home remedies. Since the seeds are believed to be health‐giving if used as part of a daily diet, they are often added to food. Black seed is widely used in obsolete medicine due to its antibacterial and anti‐inflammatory properties. For instance, it is often used to treat colds, flu, and other respiratory diseases by boiling the seeds in water and gasping the steam (Usmani and Almoselhy 2023).

Similarly, because of its antibacterial properties, black seed oil is applied to burns, wounds, and infections. Black seed is often mixed with honey or other herbs to prepare natural tonics that not only promote general health but also improve immunity. The Islamic religion is yet another field in which black seed is of immense cultural importance. It is there considered a divine gift. They quote the Prophet Muhammad (PBUH) as having said, “Use this black seed, for it is a cure for every disease except death.” Due to this, *N. sativa* is in widespread use as a medicinal plant, amulet, and sign of good health in Muslim nations globally. Black seed, as stated by Hussain and Hussain (2016), has been used as a medicinal remedy by diverse cultures for many years.

## 7 Product Development Potential of Black Seed in the Food Industry

*Nigella sativa* , or black seed as it is popularly called, has great worth in conventional medication; its gastronomical use offers similar promise. Rising consumers' interests in functional food and nutraceuticals have increased the consumption of black seed in dietaries and other food items. Especially addressed in this study are the numerous edible oils, nutraceutical, functional food, and health supplement applications of *N. sativa* in the food industry.

### 7.1 Black Seed in Functional Foods and Nutraceuticals

Individuals are becoming more interested in food items that offer extra health benefits over and above simply supplying nutrition, and from this demand, nutraceuticals and functional foods have emerged. These products serve a purpose: to treat symptoms, prevent sickness, or enhance overall health. *N. sativa* is an excellent option for this category because it is composed of various bioactive compounds that have been reported to possess antibacterial, anti‐inflammatory, antioxidant, and immune‐modulating activities (Zaky et al. 2021). Most of the health problems, including those of the immune system, cardiovascular system, and gastrointestinal system, can be managed with the consumption of functional foods containing *N. sativa* . For instance, bloating, indigestion, and other digestive problems may be assisted with dietary supplements made from black seed oil (Rahim et al. 2022). Because of its capacity to reduce inflammation, it is a perfect option for functional foods intended to treat diseases such as arthritis, heart disease, and some types of cancers (Ambati and Ramadan 2021).

Apart from its antioxidant potential, it can also serve as a natural preservative in functional food formulations. Its antibacterial properties due to thymoquinone and other substances will make food items healthier and shelf‐stable. Because more and more individuals are seeking alternatives to preserving food using chemical additives, black seed is highly demanded as an ingredient in fermented foods, dairy products, and meat products (Bashir et al. 2021) and can be incorporated into nutraceuticals that include functional foods and health supplements. The customers may include the health benefits of black seed in their daily lives by consuming these nutraceuticals. Supplements in the form of tablets, powders, or capsules are becoming highly popular and are often marketed for the ability to enhance immunity, promote heart health, and improve skin health. Due to the fact that it can be consumed in a number of forms, including oil, powder, or extracts, *N. sativa* has a broad scope of possible applications as a nutraceutical (Rahim et al. 2022).

### 7.2 Health Supplements and Edible Oils

*Nigella sativa* is primarily famous for its food supplements and edible oil, which are utilized in the food industry. Certain of the popular therapeutic solicitations of black seed oil are to endorse healthy skin, reduce allergy signs, and boost the immune system. There are numerous ways to extract the oil from the kernels, one of which is cold pressing, which conserves all the constituents. Supplements, marinades, and salad dressings all integrate *N. sativa* oil as a food element (Rahim et al. 2022). Because of the augmented mandate for functional and plant‐based oils, black seed oil is becoming progressively prevalent in the health supplement marketplace (Almatroudi et al. 2020). *N. sativa* oil is bought by numerous people due to the fact that it has great levels of omega‐3 and omega‐6 fatty acids, which are advantageous for your heart. Antioxidants in the oil lessen oxidative stress, a procedure related to age and chronic disease (Zaky et al. 2021). This has broadened the marketplace for the yonder food items and into skincare and cosmetic goods; subsequently, it is now rummage‐sale as a component in these products. To improve the digestive system, decrease inflammation, and improve the immune system, capsules with *N. sativa* powder or black seed oil are also extensively industrialized as supplements. Customers seeking to benefit from the health assistances of black seed are capable of acquiring supplements in varied forms such as capsules, pills, liquid extracts, and powder.

In accumulation, black seed oil is frequently joined with other plant extracts in medications for explicit medical circumstances like skin issues, allergic reactions, or arthritis (Ambati and Ramadan 2021). Based on Alimohammadi et al. (2013), the development of oil‐formulated *N. sativa* products has also raised the popularity of the plant among the food industry; oil is gaining further recognition as oil rich in nutrients, which can be applied in various food preparation scenarios as people seek healthier alternatives to conventional cooking oils. It may be used in food to enhance its flavor and nutritional properties along with the improvement of health because of its very powerful anti‐inflammatory and antioxidant properties (Bashir et al. 2021).

### 7.3 Applications in Novel Food Products

**FIGURE 5:**
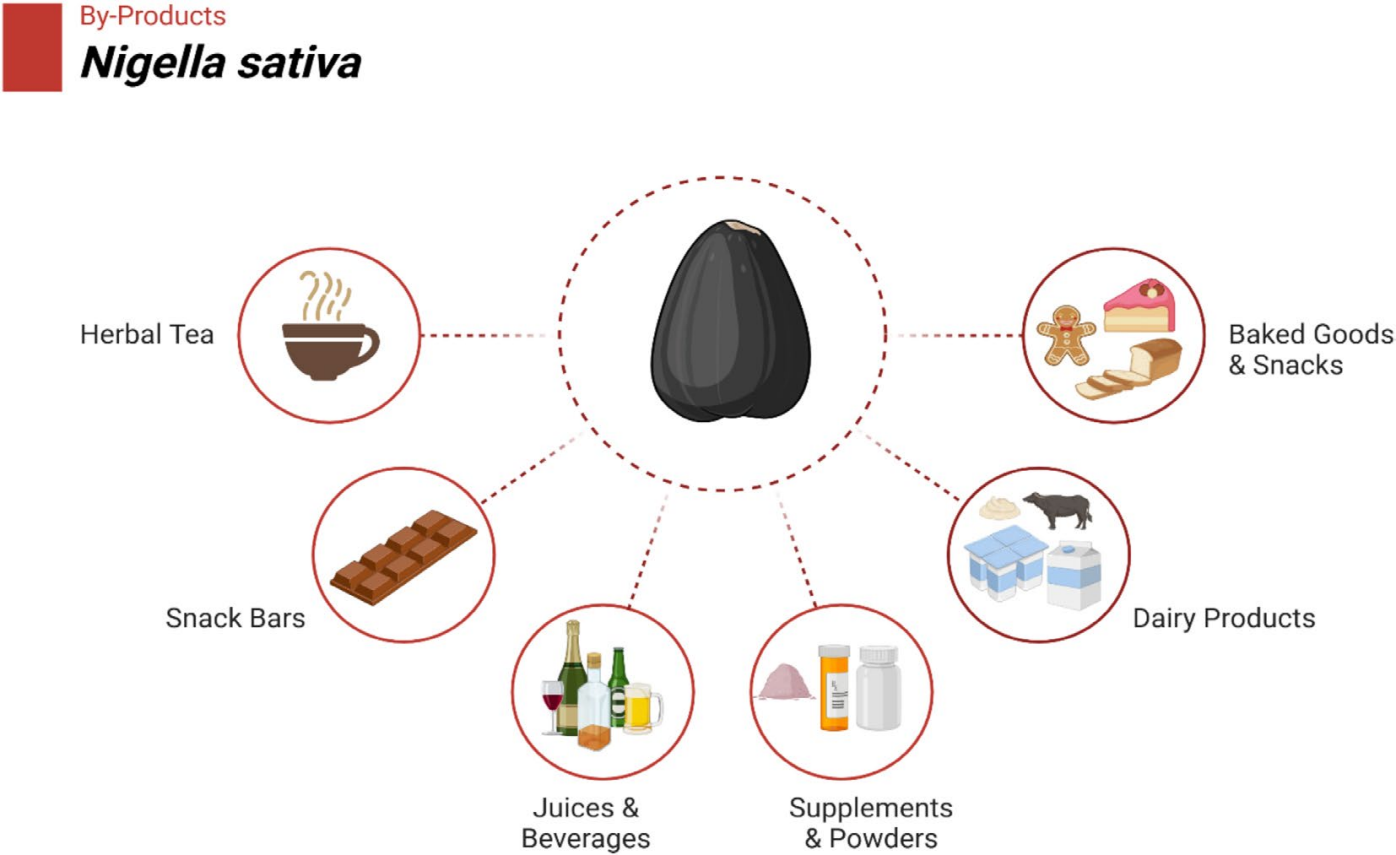
Byproducts of black seed.

Aside from these conventional uses in the development of oils and supplements, *N. sativa* is recently being explored in new food preparations. In fact, its potential as a novel material was researched recently in the utilization of black cumin seed press cake as such a novel material in developing a new generation of non‐dairy drinks such as kefir drink with grains for fermentation (Łopusiewicz et al. 2022). These drinks contain probiotics and may also have further health benefits, such as improved digestion and gut health. Another interesting application is in the preparation of functional snacks. To enhance the nutrient content of baked foods such as cookies and crackers, researchers have explored the addition of black seed flour (Figure 5). Including flour will augment these foods with additional fiber, protein, and vital fatty acids, thus appealing to health‐aware customers (Olcay and Demir 2024). Saleem et al. (2023) described that nutrient‐rich gluten‐free snacks could be manufactured by blending black seed flour with additional whole grains such as chickpeas or quinoa.

Additionally, with its role as a natural antioxidant, *N. sativa* is significantly valued for usage in functional beverage manufacturing such as smoothies and tea (Alimohamadi et al. 2014). Its anti‐inflammatory and immunostimulating effects can advantage food manufacturers in beverage fabrication, alongside augmenting the palatability of their goods (Rusmarilin et al. 2019). Functional nourishments, nutritional meals, and dietary supplements are only a scarce portion of the marketplace segments in the food sector wherein *N. sativa* is extensively promising. It is a strong agent for health advancement and disease anticipation because of its high bioactive compound concentration, comprising thymoquinone and essential fatty acids (Peng et al. 2024). While the mandate for healthier and more convenient foods upsurges, *N. sativa* can gain new solicitations in food production and preparations. Black seed will remain a critical element in generating the next cohort of functional foods and nutraceuticals because it is extremely versatile to be rummaged with edible oils, supplements, and innovative food products.

## 8 Pharmaceutical Applications of Black Seed

The therapeutic prospective of *N. sativa* , or black seed, is well recognized because of its fruitfulness in bioactive composites with therapeutic solicitations. The therapeutic value of black seed has been known for centuries, but it is only now that it is attaining momentum in up‐to‐date medicine, particularly in the expansion of novel drugs and other medicinal goods. Researchers are also seeing its probable solicitation in cosmetics and personal care products. With an emphasis on pharmaceutical composites, drug development, and cosmetic products, this investigation will discover the therapeutic solicitation of *N. sativa* .

The *N. sativa* seeds own numerous bioactive composites, comprising nigellidine, carvacrol, and thymoquinone, accountable for frequent helpful effects of the plant on health. Furthermore, the medical possessions of the plant have been well recognized. Black seed owns numerous medicinal usages, fluctuating from its anti‐inflammatory, antibacterial, and antihypertensive activities to its effects on assuaging hypertension, asthma, and diabetes (Dajani et al. 2016; Yimer et al. 2019). Natural medicine R&D for the managing of long‐lasting health problems is one of the major pharmaceutical marketplaces. Based on Zaky et al. (2021), certain *N. sativa* extracts, particularly thymoquinone, can efficiently lessen inflammation and oxidative stress. Therefore, these may be employed as therapeutic mediators to treat illnesses like arthritis, cardiovascular disease, and neurological disorders. Thymoquinone, for instance, can cure indications of rheumatoid arthritis and multiple sclerosis based on its noteworthy anti‐inflammatory as well as antioxidant possessions (Dajani et al. 2016).

*Nigella sativa* is rummage‐sale in formulations for the management of infections due to its antibacterial activity and other properties, comprising anti‐inflammatory and antioxidant activities. Black seed oil and its derivatives have potential as a natural antibiotic or antimicrobial due to their comprehensive spectrum of activity in contradiction to bacteria, fungi, and viruses (Rahim et al. 2022). Its antimicrobial and wound‐healing capabilities render it a common component in topical formulations for numerous dermatological conditions (Usmani and Almoselhy 2024).

The prospect of *N. sativa* as an adjuvant for the treatment of cancer has only newly been demonstrated. There are a number of animal experimentations that have established that the bioactive composites within black seed, such as thymoquinone, have the potential to decelerate cancer cell proliferation and could perhaps synergize with traditional chemotherapy medicines in order to improve their performance. This suggests that *N. sativa* has the potential to be rummage‐sale as an adjuvant therapy in contradiction of cancer (Ferizi et al. 2023). Pharmaceutical experts are absorbed in investigating new drug delivery systems, including self‐emulsifying drug delivery systems, which use black seed excerpts in hopes of refining the bioavailability and, therefore, the efficiency of these drugs (Halder et al. 2021).

### 8.1 Cosmetic Products and Personal Care Items

*Nigella sativa* holds immense prospective for usage in cosmetics and personal care, as well as medication, owing to its antioxidant, antibacterial, and anti‐inflammatory properties. On account of its antiaging, wound healing, and skin health refining activity, black seed oil is hugely appreciated in dermatology (Usmani and Almoselhy 2023). Due to its palliative, protective, and reformative activity on the skin, it has been found to be often used as a constituent in skin care products. *N. sativa* oil comprises elevated quantities of the essential fatty acids omega‐3 and omega‐6, believed to enhance the natural moisture and protective functions of the skin. This black seed oil is an excellent ingredient for formulations intended for dry or sensitive skin because it helps restore moisture and prevents irritation (Zaky et al. 2021).

In addition, with its antibacterial properties, the oil can be used to treat acne and other skin conditions that result from bacterial infections. Because it balances oils on the scalp and reduces inflammation, it is also used in products aimed at scalp health. This will help in treating dandruff and promote hair growth (Table 3) (Khatoon et al. 2023). In the last few years, more interest has been paid to pharmaceuticals. Due to its therapeutic properties, it became one of the key ingredients for pharmaceutical formulations. Black seed oil is claimed to be effective for wounds and scars as well as any other injury caused by physical trauma through stimulation of collagen synthesis and regeneration of skin (Dabeer et al. 2022). Its antioxidant properties reduce the visible signs of aging and promote a youthful look by protecting the skin against environmental stressors such as pollution and UV (Hussain and Hussain 2016). Conditioners and shampoos are progressively adding black seed extracts because of their positive impacts on scalp and hair growth (Alu'datt et al. 2024). The anti‐inflammatory and antioxidant properties of *N. sativa* are thought to be responsible for building a healthy scalp environment that favors hair growth. Due to its antidandruff and antifungal activities, it is very valuable in the personal care industry (Usmani and Almoselhy 2023).

**TABLE 3:** Product development potential of black seed in the food and pharmaceutical industries.

Industry	Application	Description	Example product/Research	References
Food industry	Functional foods and nutraceuticals	Black cumin seeds are rich in bioactive compounds like thymoquinone and fatty acids, making them ideal for use in functional foods	Black cumin‐based dietary supplements, smoothies, and energy bars	Zaky et al. (2021)
	Health supplements and edible oils	Black cumin oil, known for its medicinal properties, can be used in health supplements for immune support and anti‐inflammatory benefits.	*Nigella sativa* oil supplements, capsules, and infused oils for cooking	Rahim et al. (2022)
	Beverages	Black cumin seeds can create functional beverages due to their antioxidant properties and flavor profile	Black cumin tea, cold‐pressed juices, and smoothies	Łopusiewicz et al. (2022)
	Baked goods and snacks	Incorporating black cumin seeds into baked products enhances nutritional value and adds flavor	Crackers, breads, and snack bars with added black cumin seeds	Olcay and Demir (2024)
	Dairy alternatives	Black cumin seed cakes are explored as an ingredient in dairy alternatives due to their rich nutritional profile and functional properties	Plant‐based beverages such as non‐dairy yogurt or kefir made with black cumin seed meal	Łopusiewicz et al. (2022)
Pharmaceutical applications	Formulation of drugs and therapeutic agents	*Nigella sativa* has shown therapeutic potential in various diseases like diabetes, asthma, and inflammation, making it valuable in drugs	Formulations of *Nigella sativa* extracts for managing chronic conditions, such as diabetes and hypertension	Dabeer et al. (2022)
	Cosmetic products and personal care items	The antioxidant and anti‐inflammatory properties of black cumin oil make it useful in cosmetics, offering skin rejuvenation and protection	*Nigella sativa* oil‐based creams, lotions, shampoos, and serums	Hussain and Hussain (2016)
	Anti‐inflammatory agents	Black cumin seed extracts possess anti‐inflammatory properties and can be utilized in pain relief formulations	Pain relief gels or capsules containing *Nigella sativa* extracts	Yimer et al. (2019)
	Antioxidant‐rich formulations	Due to their antioxidant properties, black cumin seeds are used in formulations targeting oxidative stress and aging	Antioxidant capsules and creams, especially for antiaging treatments	Rusmarilin et al. (2019)
	Anticancer agents	Black cumin seeds contain bioactive compounds with potential anticancer effects, useful in therapeutic agents	Supplements or topical products formulated to prevent or treat cancer	Halder et al. (2021)
	Diabetes management	Black cumin seeds are considered beneficial for managing blood sugar levels due to their hypoglycemic effects	Capsules, powders, or syrups designed to aid in blood sugar regulation	Hussain and Hussain (2016)

The application of natural and herbal ingredients in cosmetics is becoming increasingly popular, and *N. sativa* is rapidly emerging as an important ingredient in organic personal care formulations. If one prefers the use of purely natural products over synthetic additives, black seed oil is an ideal choice. Based on a study (Yimer et al. 2019), it will hardly irritate or produce allergic reactions on most skin types, even the most sensitive. Its therapeutic solicitations are extensive. From its solicitation in personal care yields and cosmetics to formulating medication and therapeutic medications, black seed has a profusion of prospects to improve humanoid health and wellbeing.

## 9 Toxicity and Safe Dosage

The safety side view of *N. sativa* at therapeutic doses is usually favorable, based on investigation. The extracts, oil, and kernels of *N. sativa* demonstrated less toxicity as well as no superficial side effects at standard beneficial doses in acute animal toxicity investigations (Usmani and Almoselhy 2024). Chronic experience tests have demonstrated that *N. sativa* is not toxic when consumed over prolonged periods of time, subsidizing the indication that it is harmless to take for long‐term purposes (Dajani et al. 2016). Black seed oil, in its frequent diversities, must be consumed daily by adults in doses between 1 and 3 g (Burdock 2022). However, since the seed oil is rich in strong bioactive compounds, consuming extreme quantities of it may lead to minor toxicity or gastrointestinal turbulence. It is also crucial for persons with prevailing ailments or those on medications to consult doctors before supplementing and to use them in the exact way instructed (Mashayekhi‐Sardoo et al. 2020).

Though it is usually safe, side effects have been described in most cases, mainly when consuming the herb in surplus. Gastrointestinal disorders such as diarrhea, bloating, and nausea are some of the most common side effects experienced by patients (Mashayekhi‐Sardoo et al. 2020).

Despite being rare, allergic reactions can occur, especially in sensitive individuals, to plants within the Ranunculaceae family. Apart from this, individuals on anticoagulant medications may develop mild blood‐thinning effects due to black seed oil (Hannan et al. 2021). Some contraindications need to be considered. High doses of black seed oil are typically not recommended for pregnant women, as they may induce uterine contractions (Usmani and Almoselhy 2023). Also, patients who are sensitive to seeds or plants should be cautious while using black seed products. The immunomodulatory effects of black seed can be a contradiction with immunosuppressive drugs or treatments in individuals with autoimmune diseases.

*Nigella sativa* , also known as black seed, is a multipurpose natural product with several applications in foodstuff, medicine, and industry. Its traditional usage in Ayurveda, Unani, and Greco‐Arabic medication has been newly validated by investigations. These investigations have recognized the exact bioactive compounds thymoquinone, alkaloids, saponins, and essential fatty acids that are accountable for the comprehensive drug effects of this herb. These range from a very comprehensive assortment of activities, comprising anti‐inflammatory, antioxidant, antidiabetic, antibacterial, cardioprotective, and anticancer. Contradicting its importance in providing solutions to contemporary health matters, *N. sativa* is becoming more extensively utilized in pharmaceutical provisions, nutraceuticals, and functional foods. Notwithstanding its vast potential, its prevalent utilization is limited by numerous restrictions. Consistency is hard to attain since standardized therapeutic doses are yet to be recognized, and phytochemical confirmation differs based on manufacturing procedures. Volatility and poor absorption of thymoquinone limit its therapeutic usage, and limited information exists on the drug's long‐term safety and effectiveness. An additional factor hindering global amalgamation is regulatory differences in herbal product standards. In order to make operative usage of the drug potential of *N. sativa* , these challenges must be overcome.

*Nigella sativa* investigation holds much promise for several disciplines in the future. Accumulative consumer interest in natural remedies and advances in science are inspiring innovation in agricultural optimization by discriminating breeding and biotechnology to improve bioactive chemical fabrication. Nanotechnology and microencapsulation methods that improve the bioavailability of key ingredients like thymoquinone will assist pharmaceutical solicitations, thus possibly revolutionizing therapies for prolonged diseases. Where its anti‐inflammatory and antibacterial properties can be used technologically *N. sativa* offers intriguing prospects in functional foods, nutraceuticals, and cosmetics. To certify product consistency and safety, though, fulfillment of this promise needs uniform extraction approaches, rigorous quality control, and regulatory convergence. Extensive clinical trials are still resolutely obligatory to authenticate traditional uses on the basis of scientific indication. Successful amalgamation of *N. sativa* into modern medicine will rely on multidisciplinary collaboration among scientists, agricultural specialists, and entrepreneurs. By addressing present challenges, this ancient remedy can become the keystone of preventive medicine because it offers long‐lasting solutions for different health problems and meets the sphere demand for natural treatments.

## Author Contributions

**Muhammad Tayyab Arshad:** methodology (equal), writing – original draft (equal). **Sammra Maqsood:** data curation (equal), writing – review and editing (equal). **Ali Ikram:** supervision (equal), validation (equal). **Muhammed Adem Abdullahi:** project administration (equal), writing – original draft (equal).

## Disclosure

Institutional review board statement: This study did not involve humans or animals.

## Consent

The authors have nothing to report.

## Conflicts of Interest

The authors declare no conflicts of interest.

## Data Availability

The data supporting this study's findings are available from the corresponding author upon reasonable request.
